# A General Total Variation Minimization Theorem for Compressed Sensing Based Interior Tomography

**DOI:** 10.1155/2009/125871

**Published:** 2009-11-17

**Authors:** Weimin Han, Hengyong Yu, Ge Wang

**Affiliations:** ^1^Department of Mathematics, University of Iowa, Iowa City, IA 52242, USA; ^2^Biomedical Imaging Division, VT-WFU School of Biomedical Engineering and Sciences, Virginia Tech, Blacksburg, VA 24061, USA

## Abstract

Recently, in the compressed sensing framework we found that a two-dimensional interior region-of-interest (ROI) can be exactly reconstructed via the total variation minimization if the ROI is piecewise constant (Yu and Wang, 2009). Here we present a general theorem charactering a minimization property for a piecewise constant function defined on a domain in any dimension. Our major mathematical tool to prove this result is functional analysis without involving the Dirac delta function, which was heuristically used by Yu and Wang (2009).

## 1. Introduction

While in general an interior region-of-interest (ROI) cannot be uniquely reconstructed from projection data only associated with lines through the ROI [1, 2], in the compressed sensing framework, we recently found that a two-dimensional interior ROI can be exactly reconstructed via the total variation minimization if the function on the ROI is piecewise constant [3, 4]. The major idea behind our analysis is that the total variations of a piecewise constant function and a smooth artifact function are separable. The main mathematical tool is the expression of the two-dimensional gradient in terms of the Dirac delta function. In our analysis [3], the Delta function was instrumental but applied heuristically without mathematical rigor. In this note, we will prove rigorously a more general theorem, as an extension of the total variation minimization property presented in [3], to characterize the total variation minimization property for a piecewise constant function defined on a domain in any dimension. Such a theorem may serve as a theoretical basis for further development of interior tomography algorithms.

## 2. Theoretical Result

For piecewise constant or piecewise smooth functions, it is natural to use the space of functions of bounded variation [5] to capture the discontinuities. For an integer *d* > 0, let Ω ⊂ ℝ^*d*^ be a *d*-dimensional open bounded set and denote its boundary by ∂Ω. Then the space of functions of bounded variation is

(1) $$\text{BV}\left(\Omega \right)=\left\{\upsilon \in L^{1}\left(\Omega \right)\;|\;{||\upsilon ||}_{\text{BV}\left(\Omega \right)}<\infty \right\}.$$

It is a Banach space with the norm

(2) $${||\upsilon ||}_{\text{BV}\left(\Omega \right)}={||\upsilon ||}_{L^{1}\left(\Omega \right)}+\int_{\Omega }\left|D\upsilon \right|,$$

where ||*υ*||_*L*^1^(Ω)_ is the integral of |*υ*(*x*)| over Ω, and

(3) $$\int_{\Omega }\left|D\upsilon \right|=\sup\;\left\{\int_{\Omega }\upsilon \mathrm{div}\;\phi dx:\phi \in C_{0}^{1}{\left(\Omega \right)}^{d},\left|\phi \right|\leq 1\;\;\text{in}\;\;\Omega \right\}$$

is the total variation of the function *υ* ∈ BV(Ω). Here *C*_0_^1^(Ω) is the space of continuously differentiable functions that vanish on ∂Ω, and for *ϕ* = (*ϕ*_1_,…,*ϕ*_*d*_)^*T*^ ∈ *C*_0_^1^(Ω)^*d*^, div *ϕ* = ∑_*i* = 1_^*d*^∂*ϕ*_*i*_/∂*x*_*i*_. The Sobolev space

(4) $$W^{1,1}\left(\Omega \right)=\left\{\upsilon \in L^{1}\left(\Omega \right):\left|\nabla \upsilon \right|\in L^{1}\left(\Omega \right)\right\}$$

is a subspace of BV(Ω) and

(5) $$\int_{\Omega }\left|D\upsilon \right|=\int_{\Omega }\left|\nabla \upsilon \right|dx,\;\forall \upsilon \in W^{1,1}\left(\Omega \right).$$

We that assume Ω has a piecewise *C* ^1^ boundary, and it is decomposed into a union of a finite number of subsets with disjoint interiors

(6) $$\overset{̅}{\Omega }=\overset{M}{\underset{m=1}{⋃}}\bar{\Omega_{m}}$$

such that each subset Ω_*m*_ has a piecewise *C* ^1^ boundary. The unit outward normal vector on ∂Ω_*m*_ is denoted by *υ*_*m*_. Denote $\Gamma_{ij}=\bar{\Omega_{i}}⋂\bar{\Omega_{j}}$, which may be empty for some pairs of *i* and *j* between 1 and *M*. We write meas(Γ_*i j*_) for the (*d* − 1)-dimensional measure of Γ_*i j*_; it is the area of Γ_*i j*_ for *d* = 3, and the length of Γ_*i j*_ for *d* = 2. The symbol ∑_*i*<*j*_ will refer to a summation for those *i* and *j* with a nonempty Γ_*i j*_ in the range 1 ≤ *i* < *j* ≤ *M*. The main result of this note is the following.

**Theorem 1**

*Let *f* be a piecewise constant function corresponding to the decomposition (6):* *f*(*x*) = *c*_*m*_ ∈ ℝ for *x* ∈ Ω_*m*_, 1 ≤ *m* ≤ *M*. *Then we have*

(7) $$\begin{array}{cc} \int_{\Omega }\left|D\left(f+g\right)\right| & \;=\int_{\Omega }\left|Df\right|+\int_{\Omega }\left|\nabla g\right|\;dx,\\ & \;\;\;\;\forall g\in W^{1,1}\left(\Omega \right), \end{array}$$

(8) $$\int_{\Omega }\left|Df\right|=\underset{i<j}{\sum }\left|c_{i}-c_{j}\right|\text{meas}\left(\Gamma_{ij}\right).$$

*Consequently, we have the minimization property*

(9) $$\int_{\Omega }\left|Df\right|\leq \int_{\Omega }\left|D\left(f+g\right)\right|,\;\forall g\in W^{1,1}\left(\Omega \right).$$

**Proof**

After an integration by parts and some rearrangement, we have, for any *ϕ* ∈ *C*_0_^1^(Ω)^*d*^,

(10) $$\begin{array}{cc} \int_{\Omega }\left(f+g\right)\mathrm{div}\;\phi dx & =\overset{M}{\underset{m=1}{\sum }}c_{m}\int_{\partial \Omega_{m}}\phi \left(x\right)\cdot \upsilon_{m}\left(x\right)ds\\ & \;-\int_{\Omega }\nabla g\left(x\right)\cdot \phi \left(x\right)dx\\ & =\underset{i<j}{\sum }\left(c_{i}-c_{j}\right)\int_{\Gamma_{ij}}\phi \left(x\right)\cdot \upsilon_{i}\left(x\right)ds\\ & \;-\int_{\Omega }\nabla g\left(x\right)\cdot \phi \left(x\right)dx. \end{array}$$

By the definition (3),

(11) $$\begin{array}{cc} \int_{\Omega }\left|D\left(f+g\right)\right| & =\sup\;\{\underset{i<j}{\sum }\left(c_{i}-c_{j}\right)\int_{\Gamma_{ij}}\phi \left(x\right)\cdot \upsilon_{i}\left(x\right)ds\\ & \;\;\;-\int_{\Omega }\nabla g\left(x\right)\cdot \phi \left(x\right)dx:\phi \in C_{0}^{1}{\left(\Omega \right)}^{d},\left|\phi \right|\leq 1\;\;\text{in}\;\;\Omega \}. \end{array}$$

Taking *g*(*x*) = 0 in (11), the formula (8) follows (cf. the argument in the next paragraph for a more general situation). Moreover, from (11) again,

(12) $$\int_{\Omega }\left|D\left(f+g\right)\right|\leq \int_{\Omega }\left|Df\right|+\int_{\Omega }\left|\nabla g\right|dx.$$

For the opposite inequality, we first consider the case where $g\in C^{1}\left(\overset{̅}{\Omega }\right)$. For any *ε* > 0, define two open subsets

(13) $$\begin{array}{c} \Omega_{\varepsilon }^{\partial }=\left\{x\in \Omega :\mathrm{dist}\;\left(x,\partial \Omega \right)<\varepsilon \right\},\\ \Omega_{\varepsilon }^{\Gamma }=\left\{x\in \Omega \setminus \Omega_{\varepsilon }^{\partial }:\underset{i<j}{\min\;}\mathrm{dist}\;\left(x,\Gamma_{ij}\right)<\varepsilon \right\}. \end{array}$$

Here, dist (*x*, *D*) = min {|*x* − *y*| : *y* ∈ *D*} is the distance between *x* and a closed set *D*. Obviously, for some constant *c* > 0,

(14) $$\text{meas}\left(\Omega_{\varepsilon }^{\partial }\right)+\text{meas}\left(\Omega_{\varepsilon }^{\Gamma }\right)<c\varepsilon .$$

We start with a function $\psi_{\varepsilon }\left(x\right)\in C{\left(\overset{̅}{\Omega }\right)}^{d}$ satisfying

(15) $$\begin{array}{c} \left|\psi_{\varepsilon }\left(x\right)\right|\leq 1\;\text{for}\;\;x\in \Omega ,\\ \left|\psi_{\varepsilon }\left(x\right)\right|=0\;\text{for}\;\;x\in \Omega_{\varepsilon /4}^{\partial },\\ \psi_{\varepsilon }\left(x\right)=\frac{\mathrm{sgn}\;\left(c_{i}-c_{j}\right)\upsilon_{i}\left(x\right)}{\left|\upsilon_{i}\left(x\right)\right|}\;\text{for}\;\;x\in \Gamma_{ij}⋂\left(\Omega \setminus \Omega_{\varepsilon /2}^{\partial }\right),\\ \psi_{\varepsilon }\left(x\right)\cdot \nabla g\left(x\right)=-\left|\nabla g\left(x\right)\right|\;\text{for}\;\;x\in \Omega \setminus \left(\Omega_{\varepsilon /2}^{\partial }⋃\Omega_{\varepsilon /2}^{\Gamma }\right) \end{array}$$

and then apply the well-known mollification technique in the theory of Sobolev space [6] to define

(16) $$\phi_{\varepsilon ,\delta }\left(x\right)=\int_{B_{\delta }}\eta_{\delta }\left(x-y\right)\psi_{\varepsilon }\left(y\right)dy,$$

where *B*_*δ*_ is the ball of radius *δ* centered at the origin, *η*_*δ*_(*x*) = *η*(*x*/*δ*)/*δ* ^*d*^, and

(17) $$\begin{array}{cc} \eta \left(x\right) & =\{\begin{array}{cc} c_{0}e^{1/\left({\left|x\right|}^{2}-1\right)} & \text{if}\;\;\left|x\right|<1,\\ 0 & \text{if}\;\;\left|x\right|\geq 1, \end{array}\\ c_{0}^{-1} & =\int_{\left|x\right|<1}e^{1/\left({\left|x\right|}^{2}-1\right)}dx. \end{array}$$

Then |*ϕ*_*ε*,*δ*_(*x*)| ≤1 for *x* ∈ Ω, and for *δ* sufficiently small, *ϕ*_*ε*,*δ*_(*x*) ∈ *C*_0_^*∞*^(Ω)^*d*^. Moreover, as *δ* → 0, *ϕ*_*ε*,*δ*_(*x*) converges uniformly to *ψ*_*ε*_(*x*) for *x* ∈ Ω\(Ω_*ε*/2_^∂^ ⋃ Ω_*ε*/2_^Γ^). Thus from (11),

(18) $$\begin{array}{cc} \int_{\Omega }\left|D\left(f+g\right)\right| & \geq \underset{i<j}{\sum }\left(c_{i}-c_{j}\right)\int_{\Gamma_{ij}}^{}\phi_{\varepsilon ,\delta }\left(x\right)\cdot \upsilon_{i}\left(x\right)ds\\ & \;-\int_{\Omega }\nabla g\left(x\right)\cdot \phi_{\varepsilon ,\delta }\left(x\right)dx \end{array}$$

and as *δ* → 0, we obtain, with some constant *c*_1_ > 0

(19) $$\begin{array}{cc} \int_{\Omega }\left|D\left(f+g\right)\right| & \geq \underset{i<j}{\sum }\left(c_{i}-c_{j}\right)\int_{\Gamma_{ij}}^{}\psi_{\varepsilon }\left(x\right)\cdot \upsilon_{i}\left(x\right)ds\\ & \;-\int_{\Omega }\nabla g\left(x\right)\cdot \psi_{\varepsilon }\left(x\right)dx-c_{1}\varepsilon . \end{array}$$

Using the defining properties of *ψ*_*ε*_, we further have

(20) $$\int_{\Omega }\left|D\left(f+g\right)\right|\geq \int_{\Omega }\left|Df\right|+\int_{\Omega }\left|\nabla g\right|dx-c_{2}\varepsilon$$

for some other constant *c*_2_ > 0. Since *ε* > 0 is arbitrary, we obtain from the above relation that

(21) $$\int_{\Omega }\left|D\left(f+g\right)\right|\geq \int_{\Omega }\left|Df\right|+\int_{\Omega }\left|\nabla g\right|dx.$$

Combining (12) and (21), we conclude (7) for $g\in C^{1}\left(\overset{̅}{\Omega }\right)$.

For *g* ∈ *W* ^1,1^(Ω), we use the density of$\;\;C^{1}\left(\overset{̅}{\Omega }\right)$ in *W* ^1,1^(Ω) [6] and choose $\left\{g_{n}\right\}\subset C^{1}\left(\overset{̅}{\Omega }\right)$ such that

(22) $$g_{n}\to g\;\text{in}\;W^{1,1}\left(\Omega \right),\;\text{as}\;\;n\to \infty .$$

Since (3) defines a seminorm on BV(Ω), we have

(23) $$\left|\int_{\Omega }\left|D\left(f+g\right)\right|-\int_{\Omega }\left|D\left(f+g_{n}\right)\right|\right|\leq \int_{\Omega }\left|\nabla \left(g-g_{n}\right)\right|dx.$$

Thus, taking this limit *n* → *∞* in (7) for $g_{n}\subset C^{1}\left(\overset{̅}{\Omega }\right)$, we obtain (7) for *g* ∈ *W* ^1,1^(Ω).

As an example of (8), let Ω = *B*_*r*_0__ ⊂ ℝ^2^ be a disk of radius *r*_0_ centered at the origin. Consider a piecewise constant, radial function *f*(*r*) defined on *B*_*r*_0__ such that it has a jump *j*_*m*_ ∈ ℝ at *r*_*m*_, 1 ≤ *m* ≤ *M*, where 0 < *r*_1_ < ⋯ < *r*_*M*_ < *r*_0_. Then by (8), we have

(24) $$\int_{B_{r_{0}}}\left|Df\right|=2\pi \overset{M}{\underset{m=1}{\sum }}\left|j_{m}\right|r_{m}$$

(cf. [3, Theorem 2.2]).

## 3. Discussions and Conclusion

Some comments on the name of our approach “CS-based interior tomography” are in order. In the strict sense, compressed sensing refers to situations where the sampling scheme is built (often with random techniques) to achieve specific properties for satisfactory recovery of an underlying signal, rather than imposed by a specific detector arrangement as in limited data tomography. However, in a broad sense, compressed sensing can be interpreted as achieving better reconstruction from less data relative to the common practice. Hence, while the current name is not far off, an alternative phrase for our approach can be “total variation minimization-based interior tomography.”

 In conclusion, we have extended the total variation minimization property of a piecewise function from two-dimensions to any dimensionality in the Sobolev space, which can be used for exact reconstruction of any piecewise function on an ROI by minimizing its total variation under the constraint of the truncated projection data through the ROI. Previously, we implemented an alternating iterative reconstruction algorithm to minimize the total variation, which is time-consuming and needs improvement. Under the guidance of the theoretical finding presented here, we are working to develop a multidimensional ROI reconstruction algorithm for better performance. Clearly, major efforts are still needed in this direction.
